# Editorial: Advances in surgical techniques and ML/DL-based prognostic biomarkers for surgical and adjuvant therapies of hepatobiliary and pancreatic cancers

**DOI:** 10.3389/fonc.2026.1883725

**Published:** 2026-05-25

**Authors:** Shao-cheng Lyu, Yi Bai, Shu-ying Chen, Ren Lang

**Affiliations:** 1 Division of Hepatobiliary and Pancreatic Surgery, Department of General Surgery, Beijing Chao-Yang Hospital, Capital Medical University, Beijing, China; 2 Clinical and Research Center for Pancreatic Cancer, Capital Medical University, Beijing, China; 3 Department of Hepatobiliary and Pancreatic Surgery, The First Central Hospital of Tianjin, Tianjin, China; 4 Center for Nanomedicine, Brigham and Women’s Hospital, Harvard Medical School, Boston, MA, United States

**Keywords:** artificial intelligence, deep learning, hepatobiliary cancer, machine learning, pancreatic cancer, prognostic biomarkers, surgical techniques

## 1 Background

Hepatobiliary and pancreatic cancers remain the most aggressive and lethal malignancies globally. Despite the rapid progression in systemic anti-tumor treatment, surgery remains the primary curative option but the clinical outcome is still unsatisfying. Limited surgical resection rate and high postoperative recurrence rate are the most important factors that restricting treatment outcome, and optimizing prognostic prediction strategies, innovating surgical techniques, and developing novel adjuvant and neoadjuvant therapies are important approaches to enhancing the therapeutic efficacy of surgery.

Nowadays, continuous advancement of auxiliary examinations lays technical foundation for the precise diagnosis, classification, and prognostic prediction of hepatobiliary and pancreatic tumors. The application of machine learning (ML) models, deep learning (DL) models, and artificial intelligence (AI) in clinical practice further improves the accuracy of these predictions. The combination of these techniques enable non-invasive, high-dimensional evaluation of tumors and comprehensive data integration, and represent key future directions.

Based on above backgrounds, this Research Topic was designed to address this translational need. It brings together studies that explore how novel surgical techniques, multidisciplinary adjuvant therapies, and ML/DL/AI-driven prognostic models can enhance mechanistic understanding and support the precise implementation of individualized evaluation and treatment for hepatobiliary and pancreatic cancers.

## 2 Summarization of submitted articles

The contributing articles submitted to this Research Topic including surgical innovation, ML/DL/AI-driven prediction model establishment, multidisciplinary adjuvant therapies, and molecular biomarker discovery. Collectively, these articles offer practical insights that may enhance clinical decision-making and outcomes in the management of hepatobiliary and pancreatic cancers.

### 2.1 Surgical innovations and complication management

As the rapid development in minimal invasive technique, laparoscopic and robotic surgeries are gradually adopted in clinical practice worldwide. For distal pancreatectomy (DP), the comparison of outcomes between minimal invasive DP and traditional open DP was still limited. Wang et al. conducted a meta-analysis of propensity score-matched studies which bridge this gap. The study demonstrated that robotic DP offers significant potential advantages over open DP in reduced intraoperative blood loss, shorter hospital stays, higher spleen preservation rates, and a lower incidence of surgical site infections, highlighting the clinical utility of robotic DP. Meanwhile, Chen et al. also proposed a novel laparoscopic counterclockwise modular mesohepatectomy procedure which illustrated how to optimize the dissection sequence and utilizing Laennec’s capsule theory to overcome the technical challenge of anatomical liver resection for centrally located tumors.

Besides, researchers also focused on predicting and minimizing severe complications to improve the surgical outcome, especially for clinically relevant postoperative pancreatic fistula (CR-POPF). Zhang et al. proposed a logistic regression model including pancreatic duct diameter, pancreatic texture, CA19–9 levels, and body mass index for CR-POPF, providing a practical and effective model for CR-POPF risk assessment. Meanwhile, Ye et al. implemented a novel modified Blumgart pancreaticojejunostomy technique featuring an anterior anchoring suture approach, continuous mattress suturing technique and greater omental reinforcement. This approach not only reduced pancreatic tissue and parenchymal tearing, but also provided a physical barrier, thereby reducing the CR-POPF incidence after pancreaticoduodenectomy, particularly for high-risk patients.

### 2.2 ML/DL/AI-based modeling for precise staging and prognosis

Advanced computational tools are redefining preoperative assessments, and the submitted articles provided a series of ML/DL/AI-driven prediction model for hepatobiliary and pancreatic cancers. Chae et al. explored the capabilities of large language model (LLM) DeepSeek-R1 in preoperative T-stage discrimination for gallbladder cancer. Strikingly, the LLM analyzing unstructured radiology reports achieved high accuracy (89.6%) and outperformed traditional blood-biomarker ML models, highlighting the potential of narrative clinical data. Zhao et al. developed a nomogram integrating contrast-enhanced CT radiomics and clinical factors to predict glypican-3 (GPC3)-positive HCC. This non-invasive tool successfully identifies target populations for GPC3-directed therapies, overcoming the limitations of invasive biopsies. Furthermore, Yang et al. constructed an interpretable gradient boosting machine (GBM) model to predict overall survival for patients with large HCC following curative hepatectomy, facilitating personalized risk assessments in these patients. Yin et al. also put forward a multi-algorithm ML prognostic model for distal cholangiocarcinoma incorporating surgical features and derived neutrophil-to-lymphocyte ratio, effectively enhancing the predictive accuracy for postoperative clinical outcomes.

### 2.3 Multidisciplinary and adjuvant therapeutic strategies

Effective management of hepatobiliary and pancreatic tumor requires systemic and locoregional therapies. Ran et al. evaluated a triplet adjuvant regimen (S-1, nab-paclitaxel, and gemcitabine) for resected PDAC, reporting significantly prolonged overall and disease-free survival alongside a favorable toxicity profile compared to traditional dual therapies. This research put forward an effective chemotherapy regimen for resectable PDAC and may provide potential survival benefits for these patients. Meanwhile, two case reports also reported potential strategy for the intervention of advanced pancreatic cancer and rare pathological type of pancreatic carcinoma. In a case of advanced pancreatic cancer complicated by malignant obstructive jaundice, Li et al. demonstrated the efficacy of a sequential multidisciplinary approach—combining PTCD, a biliary seed stent, tomotherapy, and targeted-immunotherapy—to restore biliary patency and achieve sustained tumor control. Additionally, Si et al. presented a rare case of undifferentiated carcinoma with osteoclast-like giant cells of pancreas, providing a comprehensive literature review emphasizing the necessity of precise postoperative pathology and the potential role of immune checkpoint blockade in highly aggressive variants.

### 2.4 Molecular biomarkers and tumor biology

Understanding the molecular mechanism of tumor progression is essential for identifying novel therapeutic targets. Shi et al. investigated amino acid metabolic profile of HCC, identifying leucyl-tRNA synthetase 1 (LARS1) as a critical prognostic gene. Their functional analysis revealed that LARS1 knockdown significantly inhibited HCC cell proliferation, migration, and invasion *in vitro* while increasing autophagy flux, positioning LARS1 as a potentially promising therapeutic target for HCC.

## 3 Emerging themes and future directions

The contributions in this topic highlight several potential direction for future research.

Firstly, refining surgical techniques remains a cornerstone for improving postoperative outcomes. Future research should focus on optimizing current surgical technique and innovating novel minimal invasive surgical procedure in the era of laparoscopic and robotic surgery. This technical innovation should extend beyond merely improving clinical outcomes, it must aim to standardize surgical procedures and reduce technical difficulty, thereby facilitating safe adoption. Prospective and multi-center randomized trials should also be considered to validate the short-term and long-term outcome of proposed procedure.

Secondly, integrating of clinical indexes, radiomics, and multi-omics data with AI represents a major evolutionary step for clinical decision-making. Future studies should focus on developing multimodal models that can process structured clinical data, unstructured diagnostic reports, quantitative imaging features and pathological data, which will further enhance the accuracy of preoperative evaluation and personalized risk stratification. Additionally, leveraging ML/DL/AI to screen for novel predictive biomarkers represents another potential direction, which is of certain significance to improve predictive efficacy of treatment responses and prognosis.

Thirdly, improving short-term and long-term outcome of hepatobiliary and pancreatic cancer require highly optimized perioperative strategies. Future research needs to investigate the optimal sequencing, dosage, and combination of novel adjuvant and neoadjuvant therapy. Moreover, identifying reliable predictive biomarkers and prediction model to guide patient selection for these intensive regimens will also be critical to maximize survival benefits while minimizing systemic toxicity.

## 4 Conclusion

The contributing articles of this Research Topic demonstrate that advancing precision oncology for hepatobiliary and pancreatic cancers requires a multi-dimensional strategy including surgical, computational and biological advancements. These studies provide actionable insights that not only refine current clinical decision-making but also establish a solid foundation for overcoming therapeutic limitations and improving long-term patient survival in these aggressive malignancies.

